# Supplementary material for: The Gut Microbiome in Early Life Stress: A Systematic Review
Source: Nutrients. 2023 May 30;15(11):2566. doi: 10.3390/nu15112566 (PMC10255201; doi:10.3390/nu15112566)
Supplement: Supplementary file 1 [file nutrients-15-02566-s001.zip › nutrients-2373194-supplementary.pdf]

Table S1. Medline search strategy.

Adverse childhood experience strategy:

1#		"Childhood Trauma"[Title/Abstract] OR "Adolescent Trauma"[Title/Abstract] OR "Early Life Stress"[Title/Abstract] OR "Early Psychological Stress"[Title/Abstract] OR "Early Life Adversity"[Title/Abstract] OR "adverse child*"[Title/Abstract] OR "adverse adolescen*"[Title/Abstract]
2#		"adverse childhood experiences"[MeSH Terms]
3#	1# OR 2#	"Childhood Trauma"[Title/Abstract] OR "Adolescent Trauma"[Title/Abstract] OR "Early Life Stress"[Title/Abstract] OR "Early Psychological Stress"[Title/Abstract] OR "Early Life Adversity"[Title/Abstract] OR "adverse child*"[Title/Abstract] OR "adverse adolescen*"[Title/Abstract] OR "adverse childhood experiences"[MeSH Terms]
4#		"gut microbiota*"[Title/Abstract] OR "intestinal microbiota*"[Title/Abstract] OR "gastrointestinal microbiome"[MeSH Major Topic] OR "gut microflora"[Title/Abstract] OR "gastrointestinal microflora"[Title/Abstract] OR "gut microbiom*"[Title/Abstract] OR "intestinal microbiom*"[Title/Abstract] OR "intestinal microflora"[Title/Abstract]
5#	4# AND 3#	("gut microbiota*"[Title/Abstract] OR "intestinal microbiota*"[Title/Abstract] OR "gastrointestinal microbiome"[MeSH Major Topic] OR "gut microflora"[Title/Abstract] OR "gastrointestinal microflora"[Title/Abstract] OR "gut microbiom*"[Title/Abstract] OR "intestinal microbiom*"[Title/Abstract] OR "intestinal microflora"[Title/Abstract]) AND ("Childhood Trauma"[Title/Abstract] OR "Adolescent Trauma"[Title/Abstract] OR "Early Life Stress"[Title/Abstract] OR "Early Psychological Stress"[Title/Abstract] OR "Early Life Adversity"[Title/Abstract] OR "adverse child*"[Title/Abstract] OR "adverse adolescen*"[Title/Abstract] OR "adverse childhood experiences"[MeSH Terms])
6#	5# NOT "Review"	(("gut microbiota*"[Title/Abstract] OR "intestinal microbiota*"[Title/Abstract] OR "gastrointestinal microbiome"[MeSH Major Topic] OR "gut microflora"[Title/Abstract] OR "gastrointestinal microflora"[Title/Abstract] OR "gut microbiom*"[Title/Abstract] OR "intestinal microbiom*"[Title/Abstract] OR "intestinal microflora"[Title/Abstract]) AND ("Childhood Trauma"[Title/Abstract] OR "Adolescent Trauma"[Title/Abstract] OR "Early Life Stress"[Title/Abstract] OR "Early Psychological Stress"[Title/Abstract] OR "Early Life Adversity"[Title/Abstract] OR "adverse child*"[Title/Abstract] OR "adverse adolescen*"[Title/Abstract] OR "adverse childhood experiences"[MeSH Terms])) NOT "review"[Publication Type]
7#	6# AND Humans	((("gut microbiota*"[Title/Abstract] OR "intestinal microbiota*"[Title/Abstract] OR "gastrointestinal microbiome"[MeSH Major Topic] OR "gut microflora"[Title/Abstract] OR "gastrointestinal microflora"[Title/Abstract] OR "gut microbiom*"[Title/Abstract] OR "intestinal microbiom*"[Title/Abstract] OR "intestinal microflora"[Title/Abstract]) AND ("Childhood Trauma"[Title/Abstract] OR "Adolescent Trauma"[Title/Abstract] OR "Early Life Stress"[Title/Abstract] OR "Early Psychological Stress"[Title/Abstract] OR "Early Life Adversity"[Title/Abstract] OR "adverse child*"[Title/Abstract] OR "adverse adolescen*"[Title/Abstract] OR "adverse childhood experiences"[MeSH Terms])) NOT "review"[Publication Type]) AND (humans[Filter])

Anxiety strategy:

1#		"anxiety disorders"[MeSH Terms]
2#		"anxiety disorder*"[Title/Abstract] OR ("separation"[Title] AND "anxiety"[Title])
3#	1# OR 2#	"anxiety disorders"[MeSH Terms] OR ("anxiety disorder*"[Title/Abstract] OR ("separation"[Title] AND "anxiety"[Title]))

4#	3# AND all Child	("anxiety disorders"[MeSH Terms] OR ("anxiety disorder*"[Title/Abstract] OR ("separation"[Title] AND "anxiety"[Title]))) AND (allchild[Filter])
5#		"gut microbiota*"[Title/Abstract] OR "intestinal microbiota*"[Title/Abstract] OR "gastrointestinal microbiome"[MeSH Major Topic] OR "gut microflora"[Title/Abstract] OR "gastrointestinal microflora"[Title/Abstract] OR "gut microbiom*"[Title/Abstract] OR "intestinal microbiom*"[Title/Abstract] OR "intestinal microflora"[Title/Abstract]
6#	4# AND 5#	("anxiety disorders"[MeSH Terms] OR ("anxiety disorder*"[Title/Abstract] OR ("separation"[Title] AND "anxiety"[Title]))) AND ("infant"[MeSH Terms] OR "child"[MeSH Terms] OR "adolescent"[MeSH Terms]) AND ("gut microbiota*"[Title/Abstract] OR "intestinal microbiota*"[Title/Abstract] OR "gastrointestinal microbiome"[MeSH Major Topic] OR "gut microflora"[Title/Abstract] OR "gastrointestinal microflora"[Title/Abstract] OR "gut microbiom*"[Title/Abstract] OR "intestinal microbiom*"[Title/Abstract] OR "intestinal microflora"[Title/Abstract])

Trauma and Stressor Related Disorders strategy:

1#		"trauma and stressor related disorders"[MeSH Terms]
2#		"sexual abuse"[Title/Abstract] OR "child abuse"[Title/Abstract] OR "battered child"[Title/Abstract]
3#	1# OR 2#	"trauma and stressor related disorders"[MeSH Terms] OR "sexual abuse"[Title/Abstract] OR "child abuse"[Title/Abstract] OR "battered child"[Title/Abstract]
4#		"gut microbiota*"[Title/Abstract] OR "intestinal microbiota*"[Title/Abstract] OR "gastrointestinal microbiome"[MeSH Major Topic] OR "gut microflora"[Title/Abstract] OR "gastrointestinal microflora"[Title/Abstract] OR "gut microbiom*"[Title/Abstract] OR "intestinal microbiom*"[Title/Abstract] OR "intestinal microflora"[Title/Abstract]
5#	4# AND 3#	("gut microbiota*"[Title/Abstract] OR "intestinal microbiota*"[Title/Abstract] OR "gastrointestinal microbiome"[MeSH Major Topic] OR "gut microflora"[Title/Abstract] OR "gastrointestinal microflora"[Title/Abstract] OR "gut microbiom*"[Title/Abstract] OR "intestinal microbiom*"[Title/Abstract] OR "intestinal microflora"[Title/Abstract]) AND ("trauma and stressor related disorders"[MeSH Terms] OR ("sexual abuse"[Title/Abstract] OR "child abuse"[Title/Abstract] OR "battered child"[Title/Abstract]))
6#	5# NOT Review	(("gut microbiota*"[Title/Abstract] OR "intestinal microbiota*"[Title/Abstract] OR "gastrointestinal microbiome"[MeSH Major Topic] OR "gut microflora"[Title/Abstract] OR "gastrointestinal microflora"[Title/Abstract] OR "gut microbiom*"[Title/Abstract] OR "intestinal microbiom*"[Title/Abstract] OR "intestinal microflora"[Title/Abstract]) AND ("trauma and stressor related disorders"[MeSH Terms] OR ("sexual abuse"[Title/Abstract] OR "child abuse"[Title/Abstract] OR "battered child"[Title/Abstract]))) NOT "review"[Publication Type]

Child abuse strategy:

1#		"child abuse"[MeSH Terms] OR "child abuse"[Title/Abstract] OR "child neglect" [Title/Abstract] OR "child maltreatment"[Title/Abstract] OR "child mistreatment"[Title/Abstract]
2#		"gut microbiota*"[Title/Abstract] OR "intestinal microbiota*"[Title/Abstract] OR "gastrointestinal microbiome"[MeSH Major Topic] OR "gut microflora"[Title/Abstract] OR "gastrointestinal microflora"[Title/Abstract] OR "gut microbiom*"[Title/Abstract] OR "intestinal microbiom*"[Title/Abstract] OR "intestinal microflora"[Title/Abstract]
3#	1# OR 2#	("child abuse"[MeSH Terms] OR ("child abuse"[Title/Abstract] OR "child

		neglect"[Title/Abstract] OR "child maltreatment"[Title/Abstract] OR "child mistreatment"[Title/Abstract])) AND ("gut microbiota*"[Title/Abstract] OR "intestinal microbiota*"[Title/Abstract] OR "gastrointestinal microbiome"[MeSH Major Topic] OR "gut microflora"[Title/Abstract] OR "gastrointestinal microflora"[Title/Abstract] OR "gut microbiom*"[Title/Abstract] OR "intestinal microbiom*"[Title/Abstract] OR "intestinal microflora"[Title/Abstract])
4#	3# NOT Review	(("child abuse"[MeSH Terms] OR ("child abuse"[Title/Abstract] OR "child neglect"[Title/Abstract] OR "child maltreatment"[Title/Abstract] OR "child mistreatment"[Title/Abstract])) AND ("gut microbiota*"[Title/Abstract] OR "intestinal microbiota*"[Title/Abstract] OR "gastrointestinal microbiome"[MeSH Major Topic] OR "gut microflora"[Title/Abstract] OR "gastrointestinal microflora"[Title/Abstract] OR "gut microbiom*"[Title/Abstract] OR "intestinal microbiom*"[Title/Abstract] OR "intestinal microflora"[Title/Abstract])) NOT "Review"[Publication Type]

Adult Survivors of Child Adverse Events strategy:

1#		"Adult Survivors of Child Adverse Events"[MeSH Terms]
2#		"Childhood maltreatment"[Title/Abstract] OR "Childhood adversity"[Title/Abstract]
3#		"gut microbiota*"[Title/Abstract] OR "intestinal microbiota*"[Title/Abstract] OR "gastrointestinal microbiome"[MeSH Major Topic] OR "gut microflora"[Title/Abstract] OR "gastrointestinal microflora"[Title/Abstract] OR "gut microbiom*"[Title/Abstract] OR "intestinal microbiom*"[Title/Abstract] OR "intestinal microflora"[Title/Abstract]
4#	1# OR 2# OR 3#	("Adult Survivors of Child Adverse Events"[MeSH Terms] OR ("Childhood maltreatment"[Title/Abstract] OR "Childhood adversity"[Title/Abstract])) AND ("gut microbiota*"[Title/Abstract] OR "intestinal microbiota*"[Title/Abstract] OR "gastrointestinal microbiome"[MeSH Major Topic] OR "gut microflora"[Title/Abstract] OR "gastrointestinal microflora"[Title/Abstract] OR "gut microbiom*"[Title/Abstract] OR "intestinal microbiom*"[Title/Abstract] OR "intestinal microflora"[Title/Abstract])
5#	4# NOT Review	(("Adult Survivors of Child Adverse Events"[MeSH Terms] OR ("Childhood maltreatment"[Title/Abstract] OR "Childhood adversity"[Title/Abstract])) AND ("gut microbiota*"[Title/Abstract] OR "intestinal microbiota*"[Title/Abstract] OR "gastrointestinal microbiome"[MeSH Major Topic] OR "gut microflora"[Title/Abstract] OR "gastrointestinal microflora"[Title/Abstract] OR "gut microbiom*"[Title/Abstract] OR "intestinal microbiom*"[Title/Abstract] OR "intestinal microflora"[Title/Abstract])) NOT "review"[Publication Type]
